# Cellular Evidence and Source of Exosomes in the Biliary System of the Chinese Soft-Shelled Turtle, *Pelodiscus sinensis*

**DOI:** 10.3389/fphys.2019.01097

**Published:** 2019-08-27

**Authors:** Xudong Zhu, Siyi Wang, Imran Tarique, Tianci An, Huan Yang, Xuebing Bai, XiaoFei Wang, Qiusheng Chen, Ping Yang

**Affiliations:** ^1^College of Sciences, Nanjing Agricultural University, Nanjing, China; ^2^MOE Joint International Research Laboratory of Animal Health and Food Safety, College of Veterinary Medicine, Nanjing Agricultural University, Nanjing, China

**Keywords:** biliary exosomes, bile, liver, gallbladder, Chinese soft-shelled turtle

## Abstract

Exosomes are extracellular vesicles with a size of 40–150 nm that are released from a multitude of cell types and are present in biological fluids, which are particularly rich in membrane proteins. These vesicles are present in the bile, where they function as a special regulator of the digestive system; however, their source and characteristics in normal gallbladders have never been discovered. Bile, liver, and gallbladder were obtained from healthy turtles after starvation treatment. Biliary exosomes were extracted and their morphology, particle sizes, and specific proteins were characterized by transmission electron microscope (TEM), nanoparticle tracking analysis (NTA), sodium dodecyl sulfate-polyacrylamide gel electrophoresis (SDS-PAGE) staining, and Western blotting. Furthermore, immunohistochemistry and TEM were used to investigate the distribution of exosomes in both liver and gallbladder. Biliary exosomes had a round or oval shape with apparent bilayer membranes. Biliary exosomes were uniform in size, with a range from 40 to 180 nm in diameter. Immunohistochemistry showed that the CD63 exosome marker was positive and primarily distributed in the hepatocyte junction, while the free surface of gallbladder tested negative. In addition, exosomes were present in bile but absent on the apical free surface of gallbladder. This study provides direct evidences that biliary exosomes are secreted by hepatocytes into bile canaliculus and flow with bile into the gallbladder. The source and the isolated protocol of biliary exosomes may provide technical support and a theoretical basis for the exploration of potential antiviral or anti-inflammatory properties of biliary exosomes.

## Introduction

Exosomes are membrane vesicles (with a size of 40–150 nm) of endosomal origin that are released into the extracellular medium from various cells (Ding et al., 2018). Various cells can secrete exosomes; therefore, exosomes are widely present in biological fluids, such as serum (Rekker et al., 2014), breast milk (Admyre et al., 2007), sweat (Wu and Liu, 2018), and bile (Ge et al., 2017). The internal conserved proteins of exosomes include four transmembrane proteins such as CD63, heat shock proteins such as HSP70 (Théry et al., 2002), and the host protein lysosomal associated membrane protein-1 (LAMP-1) (Giri et al., 2010). Hence, CD63, HSP-70, and LAMP-1 are often used as positive biomarkers in studies of exosomes. In fact, the concentration of exosomes and their proteins are related to the physiological condition of the body. For example, the protein CD81 in serum exosomes is increased in patients with chronic hepatitis C virus (HCV) (Welker et al., 2012), and CD10 protein levels were increased in urinary exosomes of a mouse model for chronic liver injury (Conde-Vancells et al., 2010). Therefore, exosomes are considered as diagnostic and prognostic biomarkers of liver diseases (Masyuk et al., 2013). It is possible to speculate on the pathological changes of exosomes secreted by cells or organs by tracking the source of exosomes in bile.

Bile is formed by hepatocytes and modified by absorption. It flows through the bile canaliculus, and bile ducts end in the common duct (Esteller, 2008; Boyer, 2013). Depending on gallbladder contraction, the bile is released into the duodenum, where it is essential for the intestinal digestion and absorption of nutrients (Reshetnyak, 2013). Bile consists of 95% water, as well as organic and inorganic substances, and is responsible for the homeostasis of lipid metabolism, cholesterol metabolism, and the elimination of toxins (Poupon, 2012). Moreover, bile can protect animals from enteric infections (Boyer, 2013) and has anti-inflammatory properties (Li et al., 1995). Unlike mammals, the bile and gallbladder of most reptiles (such as snakes and turtles) have significant medicinal value in *materia medica*. Particularly the bile and gallbladder of turtles are used to cure hemorrhoids and anal fistula (Bensky and Gamble, 1993). Additionally, if both the bile and the gallbladder are added to wine, they have the effect of removing heat, detoxifying, and relieving cough (Teng et al., 2012). Although compositions of efficacy between bile and gallbladder have been reported (Esteller, 2008), how exosomes are secreted into bile and the role they play in bile function remain largely unknown.

Intercellular communication is one of the functions of exosomes that received much attention in the past few years (Bang and Thum, 2012; Arbelaiz et al., 2017). Previous studies showed that exosomes display a typical saucer-shaped morphology and play a major role in the biliary system (Chen et al., 2016). Currently, biliary exosomes play a significant role in the proliferation and regulatory mechanisms of cholangiocytes (Masyuk et al., 2012), inhibition of viral subgroup J (ALV-J), replication and activation of the liver immune response (Wang et al., 2014), and biliary tract diseases (Li et al., 2014). The presence of biliary exosomes has been described in chicken (Wang et al., 2014), rats (Li et al., 2017), and humans (Ge et al., 2017). However, biliary exosomes in reptiles and the source of exosomes have not been reported to date.

This study extracted the exosomes from the Chinese soft-shelled turtle bile, and investigated the morphology and source of exosomes in bile. The obtained results clarify the position of biliary exosomes during the passage of the secretory pathway in the reptilian liver, which has great significance for the exploration of the function of biliary exosomes in liver diseases.

## Materials and Methods

### Animals and Samples Preparation

Twenty Chinese soft-shelled turtles (*Pelodiscus sinensis*) weighing 500–750 g were purchased from a pond in Nanjing, China. All animals were anesthetized by intraperitoneal (IP) administration of sodium pentobarbital (20 mg/kg) and euthanized by cervical dislocation. The sample procedures were conducted according to the Animal Research Institute Committee guidelines of Nanjing Agricultural University. All the procedures was carefully performed to minimize animal suffering. The Science and Technology Agency of Jiangsu Province approved the protocol.

The turtles were sacrificed, and their livers and gallbladders were completely removed and collected. Bile was collected from gallbladder and diluted with a 0.02 M phosphate buffered saline (PBS). Furthermore, protease inhibitor was added. The bile samples were centrifuged at 1,000 rpm for 15 min at 4°C and centrifuged at 8,000 rpm for 45 min to remove tissues and impurities. Then, the supernatant fluid was collected.

### Bile Exosome Isolation

Exosomes were isolated using the Total Exosomes Isolation Kit according to the manufacturer’s instructions (Umibio Bio-technology, Shanghai, China). After the pretreatment of bile, exosome concentration solution (ECS) was added to the supernatant, and the mixed solution was stored at 4°C for 10 h, followed by centrifugation at 10,000 *g* for 1 h at 4°C. The pellet was resuspended in 0.02 M PBS, centrifuged at 12,000 *g* for 2 min at 4°C. Then, the supernatant was retained and transferred to an Exosome Purification Filter (EPF column), centrifuged at 3,000 *g* for 10 min at 4°C, and the EPF column bottom liquid was collected. In general, 50 μl of 0.02 M BPS of exosomes can be isolated from 1 ml of concentrated bile.

### Transmission Electron Microscopy and Nanoparticle Tracking Analysis

For the ultrastructural analysis, liver and gallbladder specimens were dissected and immersed in 2.5% glutaraldehyde fixative and 0.1 M PBS at 4°C for 24 h. Then, the samples were post-fixed in 1% (w/v) osmium tetroxide and washed in PBS three times. Dehydration was conducted by a graded series of ethanol (75–100%). After that, samples were soaked in propylene oxide and embedded in Araldite. Ultrathin sections of selected areas were cut, mounted on Formvar-coated grids, and stained with uranyl acetate and lead citrate for 20 min per step. The ultrastructure of oviduct parts and extracted sample were viewed by transmission electron microscopy (TEM) (Hitachi H-7650, Japan). Moreover, to analyze the distribution of particle size of the biliary exosomes, a partial sample of biliary exosomes was added to the sample cell without dilution. All operations were conducted in accordance with the instruction manual of the ZetaView^®^ NTA technique (Particle Metrix, Germany).

### Preparation and Observation of Biliary Exosome Smears

Another part of the biliary exosome sample was diluted at a dilution of 1:1 and transformed into a smear with 0.01 M PBS. Acetone was added to the smear for 10 min, exposed to distilled water for 3 min three times. The rabbit anti-CD63 (1:100) antibody (Boster Bio-technology, Wuhan, China) was used and the smear was stored at 4°C overnight. The next day, the fluorescence secondary antibody goat anti-rabbit IgG (1:5,000; Fcmacs Bio-technology, Nanjing, China) was used, and the smear was stored in the dark for 1 h followed by washing in 0.02 M PBS.

### Immunohistochemistry

Tissue sections of liver and gallbladder were stained through a standard protocol of immunohistochemistry (IHC). The tissue slides were deparaffinized in xylene three times 10 min. All slides were exposed in a graded series of ethanol (100–75%) in each grade 5 mins. Antigenic sites were exposed by boiling for 5 min in 30% sodium citrate and then rinsed three times in phosphate buffered saline (PBS). Tissue expressions were determined using the rabbit anti-CD63 (1:100; Boster Bio-technology, Wuhan, China), the rabbit anti-HSP70 (1:100; Huaan Bio-technology, Hangzhou, China), and the rabbit antibody LAMP-1 (1:100; Huaan Bio-technology, Hangzhou, China). Only PBS-incubated sections served as negative control. All the sections were stored at 4°C for whole night. The next day, we used the antibody goat anti-rabbit IgG (SABC; Boster Bio-technology, Wuhan, China) as secondary antibody. Sections were colored with DAB (1:100; Boster Bio-technology, Wuhan, China) followed by counterstaining with hematoxylin. All slides were exposed in distilled water followed by a graded series of ethanol.

### Sodium Dodecyl Sulfate-Polyacrylamide Gel Electrophoresis, Coomassie Blue Staining, and Fast Silver Staining

The concentrated bile was mixed and exosomes were extracted using the Exosomes Isolation Kit and the tissue homogenate protein of both gallbladder and liver with the lysate (RIPA Lysis Buffer). Protease inhibitor (PMSF) was added. All samples were mixed with 5 × sodium dodecyl sulfate (SDS) loading buffer at a ratio of 1:4, boiled for 10 min at 90°C, and stored at −20°C. The samples were processed with the protocol of SDS-polyacrylamide gel electrophoresis (PAGE) and Coomassie Blue staining. After SDS-PAGE, a fast liver staining kit (Biosharp bio-technology, Guangzhou, China) was used to process the samples.

### Western Blot

Western blot was performed by using equal amounts of protein (10 μg/lane) from the four samples bile, liver, gallbladder, and biliary exosomes. Samples were resuspended in 5 × SDS sample buffer, separated by SDS-PAGE. After the samples were transferred onto nitrocellulose membranes, rabbit anti-CD63 antibody (1:100; Boster Bio-technology, Wuhan, China), rabbit anti-HSP70 antibody (1:00; Huaan Bio-technology, Hangzhou, China), rabbit anti-LAMP-1 antibody (1:100; Huaan Bio-technology, Hangzhou, China), and the β-actin antibody (1:100; Boster Bio-technology, Wuhan, China) were incubated with membranes overnight at 4°C. Finally, the protein band detection was performed using an ECL detection system (Vazyme Bio-technology, Nanjing, China).

### Statistical Analysis

The Western blot data were analyzed by GraphPad Prism 7.0 software. The data are presented as mean and SEM. While the statistical significance among mean was considered difference at significant (*p* < 0.05) and highly significance (*p* < 0.01).

## Results

### Characterization of Exosomes Isolated From Turtle Bile

**Figure 1:**
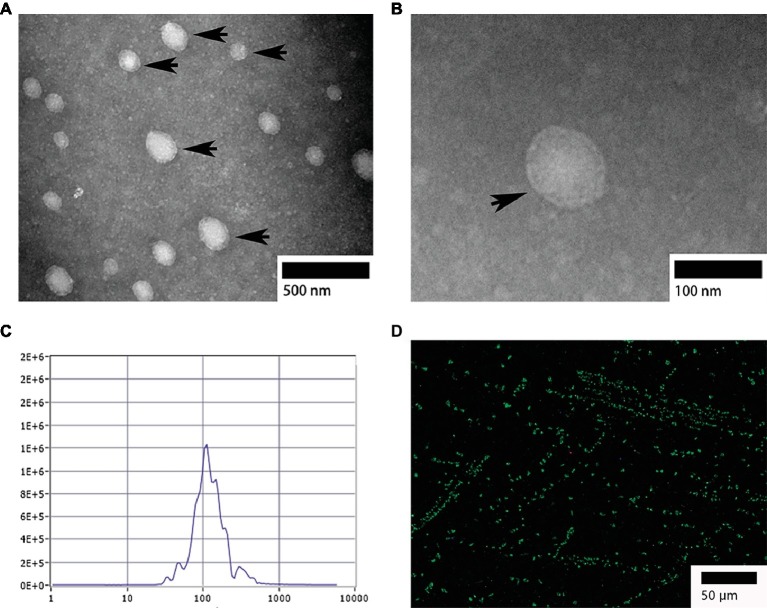
Morphological characterization of turtle biliary exosomes. **(A)** Homogeneous and round or oval-shaped exosomes dispersed at large scale; **(B)** intact bilayer membranes at small scale; **(C)** NTA for diameter distribution of turtle biliary exosomes; **(D)** CD63 immunofluorescence exosome marker in isolated bile. Bars: **(A)** 500 nm; **(B)** 100 nm; **(D)** 50 μm; biliary exosomes (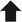).

TEM analysis showed that, the isolated exosomes from bile were round or oval in shape (Figure 1A) and formed an intact continuous bilayer membrane about 100 nm in diameter (Figure 1B). Furthermore, the size distribution of biliary exosomes was determined using nanoparticle tracking analysis (NTA). The size of biliary exosomes peaked at a mean diameter of 40–180 nm, which meets the typical morphological characteristic of exosomes (Figure 1C). About 92% of the total exosomes had a diameter of 110 nm, and the original concentration of the sample was 1.6E+7 particles/ml. To determine whether biliary exosomes can be marked *via* the exosomal protein, biliary exosomes were labeled with CD63 (green fluorescence). As shown in Figure 1D, the green fluorescence was observed by fluorescence microscopy, indicating that the biliary exosomes were marked and dispersed in a fine granular aggregate.

### CD 63, HSP-70, and LAMP-1 Expression in the Turtle Liver and Gallbladder

#### Immunohistochemistry

**Figure 2:**
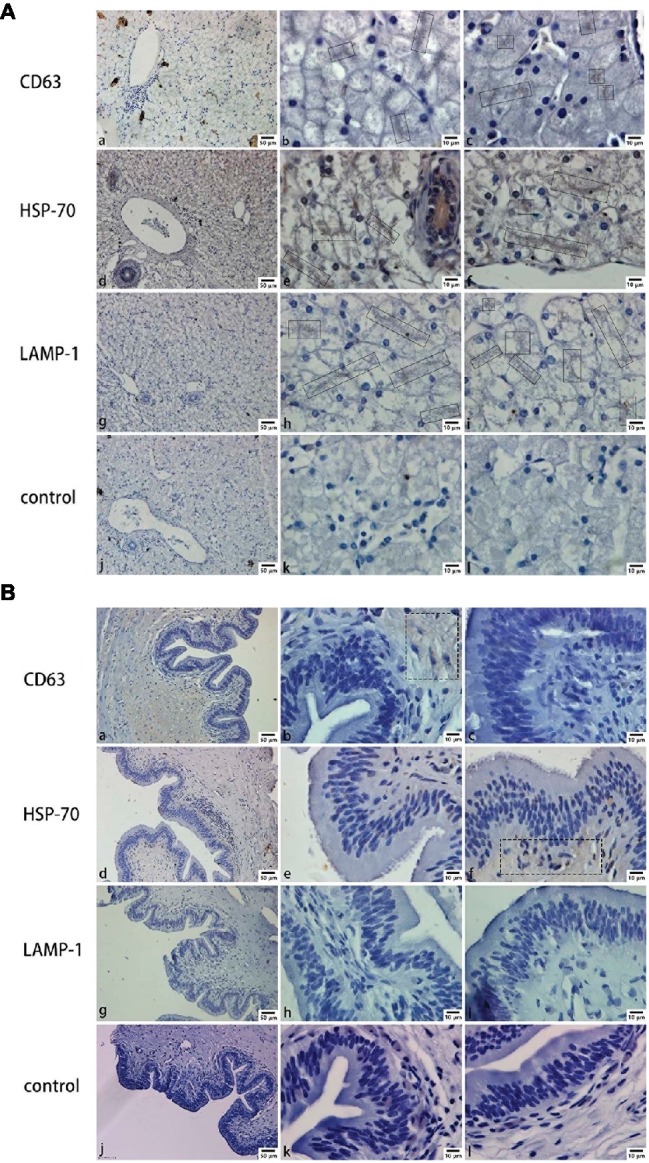
CD63, HSP-70, and LAMP-1 immunohistochemistry in both liver and gallbladder. **(A)** Exosome distribution in the liver; **(B)** exosome distribution in the gallbladder. Bars: **(a, d, g, j)** 50 μm; **(b, c, e, f, h, i, k, l)** 10 μm; rectangular boxes indicate positive expression.

Mainly positive expressions of CD63 (Figures 2Ab,Ac), HSP-70 (Figures 2Ae,Af), and LAMP-1 (Figures 2Ah,Ai) in the liver were observed around the hepatocyte junction. Most of the positive particles were assembled on the opposite side of the hepatic nucleus. However, in the gallbladder, a weakly positive expression of CD63 (Figure 2Bb) was detected in the muscular layer and a weakly positive expression of HSP-70 (Figure 2Bf) was detected in the mucosae. In contrast, negative expressions of CD63 (Figures 2Ba,Bc), HSP-70 (Figures 2Bd,Be), and LAMP-1 (Figures 2Bg,Bh,Bi) were predominantly found in mucosal epithelium and lamina propria.

### Ultrastructure and Distribution of Exosomes in the Turtle Liver and Gallbladder by Transmission Electron Microscopy

**Figure 3:**
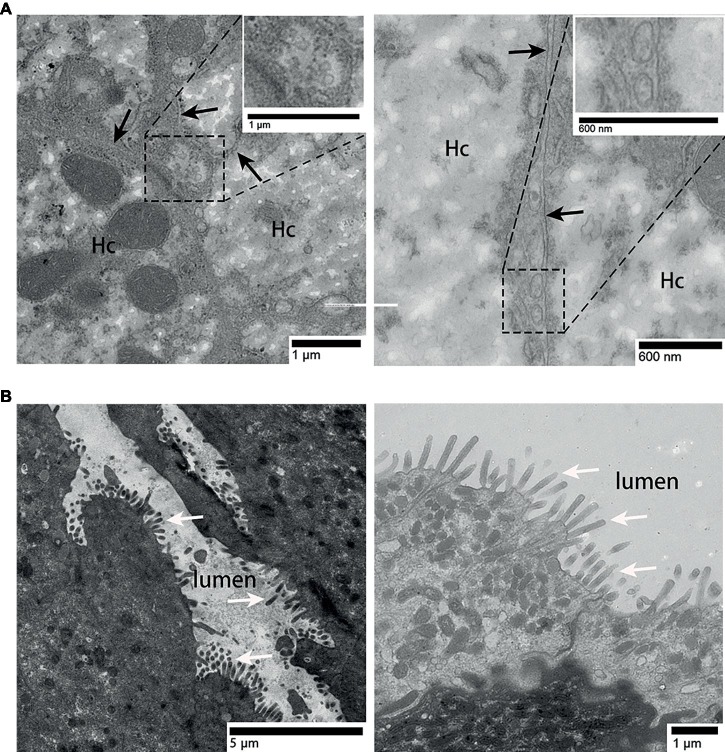
TEM photograph of gallbladder and liver tissues. **(A)** Exosomes present in the bile canaliculus; **(B)** gallbladder tissue without exosomes. Hc, hepatocyte; the junction of two hepatocyte membrane, (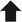); microvilli, (⇧); rectangular boxes indicate exosomes.

In the liver, hepatocyte membrane invagination formed a cavity at the junction of hepatocytes, which forms the bile canaliculus. Several exosomes were observed here (Figure 3A), the diameter of which ranged from 100 to 180 nm [Figure 3A (enlarged view)]. Different from the liver, in the surface of gallbladder lumen, no vesicle-like exosomes were observed, only many prominent microvilli were found to protrude (Figure 3B).

### Identification of Exosome Markers in the Turtle Bile, Liver, Gallbladder, and Biliary Exosomes

**Figure 4:**
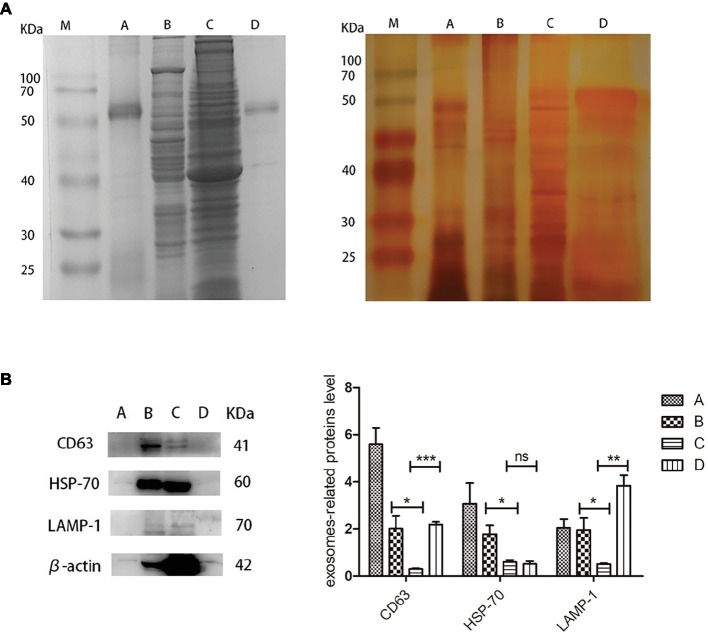
Protein analysis extracts prepared from turtle bile, liver, gallbladder, and biliary exosomes. **(A)** Coomassie Blue staining pattern (left) and silver staining pattern (right); **(B)** Western blot analysis of CD63, HSP-70, LAMP-1, and β-actin protein levels in four samples. The histogram represents the densitometric analysis of immunoblots compared to the gallbladder. M, marker; **(A)** bile; **(B)** liver; **(C)** gallbladder; **(D)** biliary exosomes; *p < 5%; **p < 1%; ***p < 0.1%; ns, not significant.

For proteomic analysis, the bile, liver, gallbladder, and biliary exosomes were separated by SDS-PAGE and stained by Coomassie Brilliant Blue and Silver staining. The pattern of Coomassie Blue staining showed one obvious but unknown protein (50–70 kDa) which corresponded to the samples of exosomes and bile (Figure 4A, left). Less bands were obtained for bile and biliary exosomes than for liver and gallbladder. Silver staining was performed to further refine the analysis of the proteins, due to its high sensitivity for the detection of low protein levels. As expected, the pattern of bands obtained for bile and biliary exosomes differed from the pattern of Coomassie Blue staining. Compared with the pattern of Coomassie Blue staining, more protein bands (<50 kDa) of bile and biliary exosomes were found as shown in Figure 4A, right. However, the proteins of liver and gallbladder barely changed. Moreover, the enrichment of exosomal marker proteins and the protein relative content were confirmed by Western blot analysis (Figure 4B). The results showed that the relative expression of CD63, HSP-70, and LAMP-1 proteins in the liver were higher than that in the gallbladder. Compared with the gallbladder, significant expression levels of CD63 and LAMP-1 were found in the biliary exosomes (Figure 4B).

## Discussion

The turtle biliary exosomes were identified by TEM and NTA. The results showed that biliary exosomes were spherical or oval in shape and had a size ranging from 40 to 180 nm. Immunohistochemistry identified different exosome markers CD63, HAP-70, and LAMP-1 in isolated bile, liver, and gallbladder. Positive signals of these exosome proteins were mainly observed between hepatocytes junctions. The biliary exosomes were further analyzed at the ultrastructural level by TEM and exosomes were found to be distributed in the bile canaliculus. Finally, Western blotting and SDS-PAGE staining were used to detect the presence and content of proteins in bile, liver, gallbladder, and biliary exosomes.

The morphological characteristics of turtle biliary exosomes were similar to that observed in human bile (Li et al., 2014). Compared with the deflated football shape of biliary exosomes from chicken (Wang et al., 2014), the isolated biliary exosomes from turtles have matching morphology, uniform particle distribution, and larger particle diameter ranging from 40 to 180 nm. Different exosome sizes might be associated with various cells of exosome origin. Isolated exosomes were also analyzed by IF to determine the presence of CD63 protein on exosomes. Due to the primitive shape of biliary exosomes, more potential physiological functions can be explored in the future research.

Previous studies have shown that vesicles are present in the lumen of intrahepatic bile ducts in mice (Masyuk et al., 2013), multivesicular bodies were found in chicken hepatocytes and the villi membrane of Disse (Wang et al., 2014; Sagredo et al., 2017). In the present study, immunohistochemical results indicated that the gallbladder may not secrete exosomes into bile, but that biliary exosomes are likely secreted by hepatocytes to the junction of hepatocytes where they then converge with bile. Furthermore, TEM clearly showed exosomes in the bile canaliculus, while no exosomes were observed in the gallbladder. Furthermore, the exosomes in bile were confirmed to be secreted by the hepatocytes into the bile canaliculus, because they were the same size (most of them are about 110 nm) as suggested under 150 nm (Patel et al., 2019). Compared with previous studies, these results indicate that part of exosomes secreted by hepatocytes are released into the space of Disse, while another part is released into the bile canaliculus, flows with the bile through cholangiocytes, and is stored in the gallbladder, then directed through the biliary system to the intestine (Wang et al., 2014). As communicator vehicle, the biliary exosomes might be involved in cellular communication of hepatocyte-to-cholangiocyte and cholangiocyte-to-hepatic sinusoidal endothelial (Masyuk et al., 2012), which requires further study. The relationship between the organs exosomes pass and the characterization of exosomes remains unknown.

Several research groups have suggested exosomes as potential screening biomarkers or therapeutic targets for diseases of the biliary tract (Sagredo et al., 2017). Recently, the concentration of extracellular vesicles was reported to be significantly elevated in bile samples from patients with malignant common bile duct (CBD) stenoses compared to controls or nonmalignant CBD stenoses (Severino et al., 2017). Therefore, exosome concentrations were lower under normal physiological conditions than under pathological conditions. In this study, biliary exosome proteins were detected on the basis of normal physiology. SDS-PAGE staining and Western blotting results support that biliary exosomes have limited proteins and lower protein content in bile. The Western blotting results of liver and gallbladder were consistent with the results of immunohistochemistry and TEM, which further corroborates that the liver may be the major organ that releases exosomes into bile. However, several exosome-associated biomarkers have been detected, and the global characterization of turtle biliary exosome proteome and bioinformatic analysis of biliary exosome proteins require further research to understand its potential function. According to the proteomics analysis of biliary exosomes, whether turtle biliary exosomes are a key component of bile function remains to be elucidated.

In conclusion, the source and secretory pathway of biliary exosomes have been studied. The extracted biliary exosomes were identified and their shape was assessed. Furthermore, a portion of exosomes secreted by the hepatocyte were released into the bile canaliculus. In general, the characteristics and presence of biliary exosomes in turtle provide a potential research platform for the study of exosomes in reptiles. This research contributes to the understanding of pathological changes of the biliary system and the potential efficacy of the reptile bile.

## Data Availability

The raw data supporting the conclusions of this manuscript will be made available by the authors, without undue reservation, to any qualified researcher.

## Ethics Statement

The sampling procedures were approved by the Nanjing Agricultural University Veterinary College. The protocol was approved by the Science and Technology Agency of Jiangsu Province. The approval ID is SYXK (SU) 2010-0005. All efforts were made to minimize the animal’s suffering.

## Author Contributions

XZ, SW, TA, HY, and XB designed and performed the experiments. XZ, SW, IT, TA, HY, XB, and XW evaluated the results. XZ, SW and IT drafted the manuscript. QC revised the manuscript and provided the professional advice. PY designed the experiments, wrote the manuscript, revised the intellectual content, and provided the professional advice. All authors read and approved the final manuscript.

### Conflict of Interest Statement

The authors declare that the research was conducted in the absence of any commercial or financial relationships that could be construed as a potential conflict of interest.

## References

[ref1] Admyre C. Johansson S. M. Qazi K. R. Filén J. J. Lahesmaa R. Norman M. (2007). Exosomes with immune modulatory features are present in human breast milk. J. Immunol. 179, 1969–1978. 10.4049/jimmunol.179.3.1969 17641064

[ref2] Arbelaiz A. Azkargorta M. Krawczyk M. Santos-Laso A. Lapitz A. Perugorria M. J. (2017). Serum extracellular vesicles contain protein biomarkers for primary sclerosing cholangitis and cholangiocarcinoma. Hepatology 66, 1125–1143. 10.1002/hep.29291 28555885

[ref3] Bang C. Thum T. (2012). Exosomes: new players in cell–cell communication. Int. J. Biochem. Cell Biol 44, 2060–2064. 10.1016/j.biocel.2012.08.007, PMID: 22903023

[ref4] Bensky D. Gamble A. (1993). Materia medica: Chinese herbal medicine. 3rd Edn. (Seattle, USA: Eastland Press), 73–78.

[ref5] Boyer J. L. (2013). Bile formation and secretion. Compr. Physiol. 3, 1035–1078. 10.1002/cphy.c120027, PMID: 23897680 PMC4091928

[ref6] Chen J. H. Xiang J. Y. Ding G. P. Cao L. P. (2016). Cholangiocarcinoma-derived exosomes inhibit the antitumor activity of cytokine-induced killer cells by down-regulating the secretion of tumor necrosis factor-α and perforin. J. Zhejiang Univ. Sci. B 17, 537–544. 10.1631/jzus.B1500266, PMID: 27381730 PMC4940629

[ref7] Conde-Vancells J. Rodriguez-Suarez E. Gonzalez E. Berisa A. Gil D. Embade N.. (2010). Candidate biomarkers in exosome-like vesicles purified from rat and mouse urine samples. Proteomics Clin. Appl. 4, 416–425. 10.1002/prca.200900103, PMID: 20535238 PMC2882112

[ref8] Ding M. Wang C. Ding M. Wang C. Lu X. L. Zhang C. P. (2018). Comparison of commercial exosome isolation kits for circulating exosomal microRNA profiling. Anal. Bioanal. Chem. 410, 1–10. 10.1007/s00216-018-1052-4 29671027

[ref9] Esteller A. (2008). Physiology of bile secretion. World J. Gastroenterol. 14, 5641–5649. 10.3748/wjg.14.5641, PMID: 18837079 PMC2748197

[ref10] Ge X. X. Wang Y. L. Nie J. J. Li Q. P. Tang L. Y. Deng X. T. (2017). The diagnostic prognostic potential and molecular functions of long non-coding RNAs in the exosomes derived from the bile of human cholangiocarcinoma. Oncotarget 8, 69995–70005. 10.18632/oncotarget.19547 29050258 PMC5642533

[ref11] Giri P. K. Kruh N. A. Dobos K. M. Schorey J. S. (2010). Proteomic analysis identifies highly antigenic proteins in exosomes from *M. tuberculosis*-infected and culture filtrate protein-treated macrophages. Proteomics 10, 3190–3202. 10.1002/pmic.200900840 20662102 PMC3664454

[ref12] Li L. Masica D. Ishida M. Tomuleasa C. Umegaki S. Kalloo A. N. (2014). Human bile contains microRNA-ladenextracellular vesicles that can be used for cholangiocarcinoma diagnosis. Hepatology 60, 896–907. 10.1002/hep.27050 24497320 PMC4121391

[ref13] Li L. Piontek K. Ishida M. Fausther M. Dranoff J. A. Fu R. (2017). Extracellular vesicles carry microRNA-195 to intrahepatic cholangiocarcinoma and improve survival in a rat model. Hepatology 65, 501–514. 10.1002/hep28735 27474881 PMC5258762

[ref14] Li Y. W. Zhu X. Y. But P. P. H. Yeung H. W. (1995). Ethnopharmacology of bear gall bladder: I. J. Ethnopharmacol. 47, 27–31. 7564418 10.1016/0378-8741(95)01249-d

[ref15] Masyuk A. I. Huang B. Q. Ward C. J. Gradilone S. A. Banales J. M. Masyuk T. V. (2012). Biliary exosomes influence cholangiocyte regulatory mechanisms and proliferation through interaction with primary cilia. Am. J. Physiol. Gastrointest. Liver Physiol. 299, 990–999. 10.1152/ajpgi.00093.2010 PMC2957333 20634433

[ref16] Masyuk A. I. Masyuk T. V. Larusso N. F. (2013). Exosomes in the pathogenesis, diagnostics and therapeutics of liver diseases. J. Hepatol. 59, 621–625. 10.1016/j.jhep.2013.03.028, PMID: 23557871 PMC3831338

[ref17] Patel G. K. Khan M. A. Zubair H. Srivastava S. K. Khushman M. D. Singh S. (2019). Comparative analysis of exosome isolation methods using culture supernatant for optimum yield, purity and downstream applications. Sci. Rep. 9:5335. 10.1038/s41598-019-41800-2 30926864 PMC6441044

[ref18] Poupon R. (2012). Ursodeoxycholic acid and bile-acid mimetics as therapeutic agents for cholestatic liver diseases: An overview of their mechanisms of action. Clin. Res. Hepatol. Gastroenterol. 36, S3–S12. 10.1016/S2210-7401(12)70015-3, PMID: 23141891

[ref19] Rekker K. Saare M. Roost A. M. Kubo A.-L. Zarovni N. Chiesi A. (2014). Comparison of serum exosome isolation methods for microRNA profiling. Clin. Biochem. 47, 135–138. 10.1016/j.clinbiochem.2013.10.020 24183884

[ref20] Reshetnyak V. I. (2013). Physiological and molecular biochemical mechanisms of bile formation. World J. Gastroenterol. 19, 7341–7360. 10.3748/wjg.v19.i42.7341 24259965 PMC3831216

[ref21] Sagredo A. I. Sepulveda S. A. Roa J. C. Oróstica L. (2017). Exosomes in bile as potential pancreatobiliary tumor biomarkers. Transl. Cancer Res. 6, S1371–S1383. 10.21037/tcr.2017.10.37

[ref22] Severino V. Dumonceau J. M. Delhaye M. Moll S. Annessi-Ramseyer I. Robin X. (2017). Extracellular vesicles in bile as markers of malignant biliary stenoses. Gastroenterology 153, 495–504.e8. 10.1053/j.gastro.2017.04.043 28479376

[ref23] Teng Y. Zhang S. L. Wang C. L. (2012). The technology study on the series products of *Trionyx sinensis*. Acad. Period. Farm Prod. Proc. 4, 119–121.

[ref24] Théry C. Zitvogel L. Amigorena S. (2002). Exosomes: composition, biogenesis and function. Nat. Rev. Immunol. 2, 569–579. 10.1038/nri855 12154376

[ref25] Wang Y. Wang G. H. Wang Z. Z. Zhang H. G. Zhang L. Cheng Z. Q. (2014). Chicken biliary exosomes enhance CD_4_^+^ T proliferation and inhibit ALV-J replication in liver. Biochem. Cell Biol. 92, 145–151. 10.1139/bcb-2013-0096 24697699

[ref26] Welker M. W. Reichert D. Susser S. Sarrazin C. Martinez Y. Herrmann E. (2012). Soluble serum CD81 is elevated in patients with chronic hepatitis C and correlates with alanine aminotransferase serum activity. PLoS One 7:30796. 10.1371/journal.pone.0030796 PMC3280260 22355327

[ref27] Wu C. X. Liu Z. F. (2018). Proteomic profiling of sweat exosome suggests its involvement in skin immunity. J. Invest. Dermatol. 138, 89–97. 10.1016/j.jid.2017.05.040 28899687

